# Exploring the Frontiers of Virus–Host Interactions—3rd Edition

**DOI:** 10.3390/v16101544

**Published:** 2024-09-30

**Authors:** Anupam Mukherjee, Parikshit Bagchi

**Affiliations:** 1 Division of Virology, ICMR-National Institute of Translational Virology and AIDS Research, Pune 411026, India; 2 Department of Molecular Microbiology, Washington University School of Medicine in St. Louis, St. Louis, MO 63110, USA

It is with great enthusiasm that we introduce the third edition of the “Virus–Host Interaction” series, a collection that epitomizes the ever-evolving landscape of virology. The 12 research articles included in this edition represent a diverse array of studies, each contributing crucial insights into the intricate dynamics between viruses and their host organisms. As our understanding of these interactions deepens, so too does our capacity to develop innovative strategies for preventing, diagnosing, and treating viral infections that continue to pose significant threats to global health.

This Issue opens with Harford’s perspective on the ongoing advancements in antiviral therapies, setting the stage for the comprehensive exploration of virus–host interactions that follows [1]. In an era where the rapid emergence of drug-resistant viral strains challenges existing treatment modalities, Harford’s article emphasizes the critical need for continued innovation in antiviral drug development. By highlighting both the successes and limitations of current therapies, this piece serves as a compelling call to action for the scientific community to push the boundaries of what is possible in antiviral research.

Fan et al. follow with a timely and in-depth investigation into the molecular mechanisms underlying SARS-CoV-2 infection [2]. As the causative agent of the COVID-19 pandemic, SARS-CoV-2 has brought unprecedented attention to the field of virology. This study probes into the virus’s complex interactions with host cellular machinery, revealing potential therapeutic targets that could pave the way for more effective treatments. By elucidating the molecular pathways that contribute to the virus’s pathogenicity, Fan et al. provide a foundation for future research aimed at mitigating the impact of this and similar coronaviruses. In a related study, Franco et al. evaluate the antibody response in Sputnik V vaccine recipients in Venezuela during the circulation of the Gamma variant. They report that while Sputnik V-induced antibodies can recognize and neutralize multiple SARS-CoV-2 variants, the Omicron variant exhibits the highest degree of immune escape, highlighting the ongoing challenge of variant-specific vaccine effectiveness [3]. Both studies underscore the importance of continued efforts to understand and combat the evolving threat posed by SARS-CoV-2 and its variants.

The zoonotic potential of viruses is another critical area of focus in this edition. Li et al.’s examination of the H4N6 avian influenza virus underscores the ongoing risk posed by zoonotic pathogens [4]. Their study of the virus’s epidemiology and pathogenicity not only highlights the importance of surveillance in preventing potential outbreaks but also emphasizes the need for a deeper understanding of the factors that enable cross-species transmission. As zoonotic viruses continue to emerge, Li et al.’s findings serve as a crucial reminder of the interconnectedness of animal and human health.

Building on the theme of virus-induced host responses, Coelho et al. explore the role of the kallikrein–kinin system (KKS) in viral infections [5]. Their research sheds light on the intricate interplay between viral pathogens and the host’s inflammatory response, with a particular focus on the KKS’s involvement in mediating inflammation. By identifying this system as a potential therapeutic target, Coelho et al. open new avenues for the development of treatments that could modulate the host’s immune response to viral infections, thereby reducing disease severity and improving patient outcomes.

The complexity of immune responses to viral infections is further explored in two studies focusing on the Dengue virus [6,7]. Shivaprasad et al. investigate the role of T cells and cytokines in the severity of Dengue virus infection, providing valuable insights into the mechanisms that drive immune-mediated pathology. Their findings have significant implications for the development of vaccines and therapeutic strategies that aim to enhance protective immunity while minimizing harmful immune responses. Santana and Gomez, on the other hand, examine the genetic diversity of the Dengue virus and its implications for vaccine development. Their study highlights the challenges posed by viral genetic variability and underscores the need for vaccines that can provide broad protection across different viral strains.

Viral evasion of the host immune response is a recurring theme in this edition, with Ni et al. providing a comprehensive analysis of reovirus–host interactions [8]. Their study reveals how a reovirus can evade immune detection by manipulating host immune pathways, offering new insights into the mechanisms that enable viruses to persist in the host. This research not only enhances our understanding of reovirus pathogenesis but also provides a model for studying viral immune evasion strategies more broadly.

The challenges of developing effective treatments for neurotropic viruses are exemplified in Ashraf and Uversky’s review of the rabies virus [9]. Rabies, with its devastating neurological impact, remains a significant public health concern in many parts of the world. This review meticulously details the molecular mechanisms that underlie rabies virus infection, highlighting the difficulties in treating this lethal virus once symptoms have appeared. The authors discuss the ongoing efforts to develop more effective treatments and vaccines, emphasizing the importance of early diagnosis and post-exposure prophylaxis in managing rabies infections.

Liparulo and Shoemaker address another significant challenge in virology: the continuous evolution of influenza viruses. Their study focuses on the mechanisms of antigenic drift and shift, which enable influenza viruses to evade immunity and necessitate the frequent updating of vaccines [10]. By exploring the factors that drive influenza virus evolution, Liparulo and Shoemaker provide critical insights that could inform the development of more effective, broadly protective vaccines. Their work underscores the ongoing challenge of staying ahead of a virus that is constantly adapting to its environment.

The potential for novel therapeutic strategies is also highlighted in this edition, with Lebeau et al. exploring the antiviral potential of PolyIC [11]. As a synthetic analog of double-stranded RNA, PolyIC has been shown to stimulate innate immune responses and has potential applications in combination therapies for viral infections. Lebeau et al.’s study investigates the efficacy of PolyIC in enhancing antiviral immunity, providing a promising avenue for future research into combination therapies that could improve the effectiveness of existing antiviral treatments.

Dass et al.’s study on Herpes Simplex Virus Type 2 (HSV-2) further extends the scope of this edition by exploring how HSV-2 manipulates the host autophagy pathway through the interferon signaling pathway to enhance its survival and replication [12]. Their research investigates the complex interplay between the virus and the host immune response, revealing potential therapeutic targets within the autophagy and interferon pathways that could be exploited to combat HSV-2 infections. This study underscores the importance of understanding virus–host interactions at the molecular level, particularly how viruses can hijack cellular processes to facilitate their own survival.

Finally, Lo et al. explores the role of deoxynucleoside triphosphates (dNTPs) in viral replication, offering new insights into potential therapeutic targets. Their research highlights the critical role of dNTP availability in the replication of viral genomes and suggests that targeting dNTP synthesis could be an effective strategy for inhibiting viral proliferation [13]. This study not only enhances our understanding of the molecular underpinnings of viral replication but also identifies a novel target for the development of antiviral therapies.

As the editors, we are delighted to present these 13 papers, each of which represents a significant contribution to the field of virology. Together, they reflect the breadth and depth of research being conducted on virus–host interactions, from the molecular mechanisms of viral infection to the development of novel therapeutic strategies. The insights and discoveries presented in this edition underscore the importance of continued research in virology, not only to address the challenges posed by current viral threats but also to prepare for the emergence of new pathogens in the future.

We would like to extend our sincere gratitude to the authors, reviewers, and the editorial team for their hard work and dedication in bringing this edition to fruition. Their contributions have been instrumental in advancing our understanding of virus–host interactions and in paving the way for future research and innovation in this critical field.

As we continue to navigate the complexities of viral infections and their impact on global health, we are confident that the research presented in this edition will inspire further advancements in the fight against viral diseases. We look forward to seeing how these findings will contribute to the development of new strategies for preventing, diagnosing, and treating viral infections, ultimately improving health outcomes for individuals and communities around the world.
